# T Regulatory and T Helper 17 Cells in Primary Sjögren's Syndrome: Facts and Perspectives

**DOI:** 10.1155/2015/243723

**Published:** 2015-04-28

**Authors:** Alessia Alunno, Francesco Carubbi, Onelia Bistoni, Sara Caterbi, Elena Bartoloni, Giulia Mirabelli, Francesca Cannarile, Paola Cipriani, Roberto Giacomelli, Roberto Gerli

**Affiliations:** ^1^Rheumatology Unit, Department of Medicine, University of Perugia, 06123 Perugia, Italy; ^2^Rheumatology Unit, Department of Biotechnological and Applied Clinical Sciences, University of L'Aquila, 67100 L'Aquila, Italy

## Abstract

Historically, primary Sjögren's syndrome (pSS) was thought to be a T helper (h) 1 driven disease due to the predominance of CD4^+^T lymphocytes and their products in target organs and peripheral blood of patients. In the last decades, the identification of a number of T cell subsets, including Th17, T regulatory (Treg), and follicular helper T cells, challenged this long-standing paradigm and prompted to identify their role in pSS pathogenesis. In addition the impact of abnormal proinflammatory cytokine production, such as IL-6, IL-17, IL-22, and IL-23, has also attracted considerable attention. However, although several studies have been carried out in experimental models and patients with pSS, many aspects concerning the role of Treg cells and IL-17/Th17 cell system in pSS pathogenesis are not fully elucidated. In particular, the role played by different IL-17-producing T cell subsets as well as the effects of pharmacological therapies on Treg/Th17 cell balance represents an intriguing issue. The aim of this review article is to provide an overview of current knowledge on Treg cells and IL-17-producing T cells in pSS pathogenesis. We believe that these insights into pSS pathogenesis may provide the basis for successful therapeutic intervention in this disease.

## 1. Introduction

Primary Sjögren's syndrome (pSS) is an autoimmune disease with exocrine gland dysfunction and at least one-third of patients experience multiorgan involvement [1]. Furthermore, 5% of patients may develop lymphoma, mainly the mucosa-associated lymphoid tissue (MALT) non-Hodgkin lymphoma (NHL), which represents the most severe complication of the disease [2].

Histologically, pSS is characterized by extensive target tissue infiltration of lymphocytes, mainly represented in the salivary glands by T cells, predominantly CD4^+^T cells, but also CD8^+^T cells [3]. Although T cells predominate in mild lesions, B cells are the most represented cell subset in the advanced lesions, with a decreased percentage of macrophages and an increased percentage of dendritic cells [4–6]. Infiltrating lymphocytes are often organized into tertiary ectopic lymphoid structures, showing a network including specific segregated T- and B-cell zones, associated with follicular dendritic cells, with a specific glandular cytokine profile [7]. Despite the presence, and sometimes predominance, of T cells in salivary gland infiltrates, their pathogenic role in pSS remains to be elucidated.

CD4^+^T helper (Th) lymphocytes have been long known to be distributed into Th1 and Th2 cells, based on distinct cytokine patterns [8]. An imbalance between type 1 cytokine-producing Th1 cells and type 2 cytokine-producing Th2 cells has been considered as predisposing to autoimmunity. Historically, pSS was thought to be a Th1 driven disease due to the predominance of CD4^+^T lymphocytes and their products, namely, interferon-*γ* (IFN-*γ*), in target organs and peripheral blood (PB) of these patients. Inevitably, on the basis of* in vitro* and* in vivo* observations, the role of Th1 and Th2 cells in pSS has become contradictory. In the last decade, a number of Th cell lineages, including Th0, Th17, regulatory T (Treg), and follicular helper T (Tfh) cells, have been identified [9]. This challenged the long-standing paradigm of a Th1/Th2 immune response and prompted to identify their role in the pathogenesis of autoimmune diseases including pSS. In particular, Th17 cells were described and IL-17 was acknowledged as a prime representative of the new generation of proinflammatory cytokines [10]. Concomitantly, regulatory T (Treg) cells were identified as a unique population of Th cells that restrain excessive activation of effector lymphocytes [11].

Besides the role of different cell subsets in pSS pathogenesis, the impact of abnormal cytokine production, such as IL-6, IL-17, and BAFF, has also attracted considerable attention. In particular, it is a challenge to understand how the interaction between several interconnected networks of cytokines impact so many different cell populations, on one hand, and how the interplay of cytokine-producing T and B cells shifts the balance towards autoreactive T and B lymphocytes, on the other.

**Figure 1:**
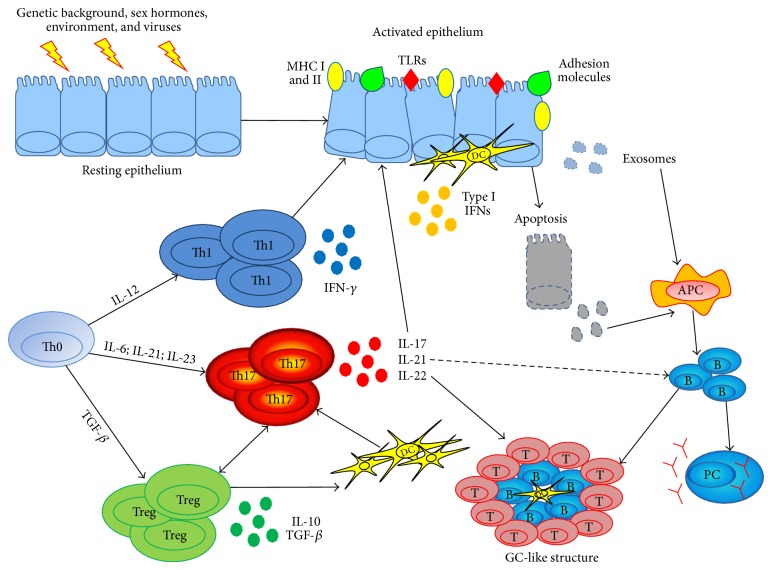
Cellular and molecular players in the pathogenesis of primary Sjögren's syndrome. MHC = major histocompatibility complex, TLR = toll-like receptor, DC = dendritic cell, Th = T helper cell, IFN = interferon, IL = interleukin, APC = antigen presenting cell, Treg = T regulatory cell, PC = plasma cell, GC = germinal center, TGF = transforming growth factor.

The ongoing progress in discovering lymphocyte subsets and the lengthening list of cytokines involved has further fuelled the debate on pSS pathogenesis (Figure 1). The main purpose of this review is to summarize and highlight the role of IL-17-producing T cells and Treg cells in pSS pathogenesis, offering the rationale for new therapeutic approaches in this disease.

## 2. Regulatory T Cells in pSS

Treg cells were initially identified in mice and humans according to the high surface expression of the alpha chain of IL-2 receptor (IL-2R*α*, CD25) and the capability to prevent polyautoimmunity in an experimental animal model [11]. This cellular subset exerts suppressive activity towards autoreactive lymphocytes via either cell-cell contact or the release of soluble mediators including IL-10 and transforming growth factor *β* (TGF-*β*) [12]. The commitment of a naïve T lymphocyte towards a Treg phenotype is dependent of a peculiar cytokine microenvironment and of the expression of the forkhead box protein P3 (FoxP3) transcriptional factor, which ensures Treg suppressive function and represents to date the most specific Treg marker [13, 14]. Intriguingly, soluble mediators required for Treg commitment are also those which participate in the commitment of a pathogenic IL-17-producing T helper (Th) subset, the Th17 cells. In fact, TGF-*β* is required in both cases, but the concurrent presence or absence of IL-6 leads to the generation of either Th17 or Treg cells, respectively [15]. It is evident, therefore, that such a fine balance between these two cell subsets may be easily disturbed leading to a predominance of pathogenic cells and therefore to the development of autoimmunity. In this context, it has been demonstrated that a committed Treg cell can be turned into a Th17 cell in the presence of appropriate stimuli. An interesting study, employing a FoxP3 reporter mouse, revealed that the blockade of indoleamine 2,3-dioxygenase, a master regulator of self-tolerance, in the presence of IL-6 induced conversion of Treg into Th17-like cells in rodent tumor-draining lymph nodes [16].

As far as the role of Treg cells in pSS pathogenesis is concerned, ten studies have been published and the results are often controversial [17–26]. Therefore, similar to systemic lupus erythematosus (SLE) and rheumatoid arthritis (RA), conclusive data are still lacking [27, 28] (Table 1).

Such discrepancies may be explained at least in part by the different strategies employed to assess Treg cells over time. Generally, the approach of earlier studies was to enumerate the proportion of circulating Treg cells according to the high surface expression of CD25. Subsequently, however, the coexpression of FoxP3, the most specific marker of Treg cells, was also evaluated.

Five studies reported an overall reduction of PB CD25^high^Treg cells [18, 19, 22, 24, 26], but, of note, an association between this reduction and clinical or serological features was observed only in 2 studies. In detail, Liu et al. reported an inverse correlation between the percentage of Treg cells and C reactive protein, erythrocyte sedimentation rate, rheumatoid factor, and immunoglobulin (Ig) G concentration [19]. Conversely, Szodoray et al. described that Treg cell reduction in the PB was more pronounced in patients with a milder clinical picture not presenting extraglandular manifestations [22].

In striking contrast, two studies reported an increase of circulating Treg cells in pSS, not associated, however, with any clinical or serological features [17, 23] and three described that PB CD4^+^CD25^high^ cell percentages were similar in pSS and controls [20, 21, 25].

Only two studies tried to correlate the disease activity with the percentage of peripheral Treg cells. In particular, we subdivided patients into two groups according to the EULAR Sjogren's syndrome disease activity index (ESSDAI) values: patients with ESSDAI ≤2, whose only manifestation of disease was a mild stable polyclonal hypergammaglobulinemia, were considered inactive, whereas patients with ESSDAI >2 were classified as active [26]. We observed that the disease activity did not influence the number of circulating CD4^+^CD25^high^Treg cells. Similarly, another study defined clinical active disease as the presence of one or more glandular and extraglandular features recognized by the ESSDAI and the Sjögren syndrome disease activity index (SSDAI) with the exception of fatigue and serologic activity as the presence of increased serum viscosity, elevated IgG, IgM, IgA, or decreased levels of complements C3 and C4 [25]. No statistically significant differences in the frequency of CD4^+^CD25^high^FoxP3^+^ cells were found.

The apparently paradoxical increase as well as the normal values of circulating Treg cell percentages described in some studies deserves some consideration. The surface expression of CD25 in not limited to Treg cells but can be shared by recently activated lymphocytes [29]. In this setting, Han et al. observed that only a subgroup of CD25^high^ T cells coexpresses FoxP3 in RA patients [30]. Therefore, higher percentages of CD25^high^ T cells in RA patients may be due more likely to a contamination of recently activated cells rather than to an increase of real Treg cells. In line with this hypothesis, Sarigul et al. reported that, besides an increase of circulating CD25^high^ Treg cells in pSS, the proportion of FoxP3^+^ cells in the PB was comparable to that of normal subjects and patients with RA [23].

In addition, unlike murine CD25^high^ Treg cells, those isolated from humans include different cell subsets that, although developmentally related, display different phenotype and function. On this basis, in 2011, Miyara and Sakaguchi suggested that the assessment of CD45RA on the cell surface and of Helios transcription factor may help to distinguish Treg cell subsets with consistent suppressive activity. Unfortunately such approach was not employed in the majority of studies investigating Treg cell in pSS; hence several issues concerning the reproducibility and comparability of different studies remain open [31].

Finally, it has been recently put forward the hypothesis that CD25 expression is not mandatory to confer a regulatory phenotype [32]. In this setting, recent studies identified a T lymphocyte subpopulation expressing FoxP3, but lacking CD25 surface molecule, that is expanded in the PB of patients with SLE [33, 34]. However, the lack of a consistent suppressive activity exerted by CD25^−^FoxP3^+^ cells underscored the need to identify more specific Treg cell markers to be combined with FoxP3. Translating the knowledge on murine Treg cells to humans, the glucocorticoid induced tumor necrosis factor receptor related protein (GITR), a well-characterized marker of Treg cells in mice, gained growing scientific interest [35]. In fact, GITR and FoxP3 coexpression allows identifying T cells with regulatory phenotype and function independently of CD25 expression in normal subjects. Of interest, GITR blockade abrogates their suppressive activity suggesting that this molecule is involved in conferring regulatory properties [36].

Moreover, a functionally suppressive CD25^low/−^GITR^+^ cell subset was found to be expanded in the PB of patients with SLE or pSS and this expansion was associated with a reduction of conventional CD25^high^ Treg cells [26, 37]. Intriguingly, the expansion of CD25^low/−^GITR^+^ cell was more pronounced in patients with inactive disease suggesting a certain attempt to rebalance the reduction of conventional Treg cells that is working, at least, in milder disease.

Moving to tissue level, the evaluation of Treg cells in pSS minor salivary glands (MSGs) was performed in four studies [18, 21, 23, 26]. Li et al. observed a reduced number of CD25^+^ cells in pSS-MSGs compared to those with nonautoimmune parotitis [18]. However, taken the fact that also activated cells may express CD25, these observations do not allow drawing any definitive conclusion on the presence of Treg in pSS-MSGs. Therefore, FoxP3 has been subsequently analyzed with either immunohistochemistry [18, 21, 23, 26] or polymerase chain reaction [18], showing a consistent expression of this transcription factor and supporting the presence of Treg cells in pSS-MSG mononuclear cell infiltrate. Of note, however, both CD25^+^ and CD25^−^ cells were included in the FoxP3^+^ area within the salivary gland infiltrate, and some of them coexpressed GITR, suggesting the presence of not only conventional CD25^high^, but also the aforementioned CD25^low/−^GITR^+^ suppressive cell subset in MSG during pSS [26].

Of particular interest, an attempt to correlate FoxP3 staining, namely the amount of infiltrating Treg cells, and the extent of glandular involvement was also pursued in two studies [21, 23]. A direct correlation between FoxP3 staining and either Sarigul et al. [23] focus score or Tarpley's score [21] was observed. These findings, concerning the correlation between the amount of infiltrating Treg cells and the severity of tissue inflammation, are in line with those obtained in rheumatoid synovium and point out the role of Treg cells in counteracting local tissue inflammation that, however, may be ineffective [38, 39].

In fact, although the* in vitro* functional assays performed in four studies pointed out that the suppressive activity of PB CD25^high^ cells seems preserved in pSS [17, 18, 22, 26], it is not possible to ascertain whether Treg cells are able to exert their suppressive activity* in vivo* or they are affected by local inflammatory microenvironment.

## 3. IL-17-Producing T Cells in pSS

IL-17 is a family of cytokines including six members, from A to F, with a wide range of biological activities [40]. Besides physiological processes, such as host defense against microbial infections, IL-17 is a leading actor in pathologic conditions, including cancer and autoimmune disorders, due to a strong proinflammatory potential [41]. In fact, upon binding to its receptor, IL-17 triggers downstream events culminating in the transcription of proinflammatory genes, such as cytokines, chemokines, and matrix degrading enzymes, by target cells [10]. Although the main cellular source of IL-17 is represented by T lymphocytes, growing evidence suggests that also neutrophils and mast cells participate in the balance of this cytokine [42, 43].

The commitment of a naïve CD4^+^T lymphocyte towards a T helper (Th) 17 cell occurs in the presence of a peculiar cytokine milieu. Indeed, the upregulation of the retinoic acid orphan receptor (ROR) *γ*t transcription, the expression of IL-17, and the stabilization of the Th17 phenotype require the concurrent presence of IL-6, TGF-*β*, IL-21, IL-1*β*, and IL-23. Besides IL-17, Th17 cells are able to produce also IL-21 and IL-22. IL-21 collaborates with dendritic cell-derived TGF-*β* to amplify the tendency to Th17-cell differentiation and induces these lymphocytes to express receptors for IL-23. The latter cytokine is required for the maintenance of Th17 phenotype [15].

In addition, it has been recently reported that a subset of Th17 cells, as identified by the coexpression of CCR6 and CXCR3, is able to produce also IFN-*γ* [44]. Taken the well-established role of IFN-*γ* in the pathogenesis of autoimmune diseases, including pSS, this intriguing finding further underscores the relevance of Th17 cells in such scenario.

It is interesting that, at least in mice, Th17 lymphocytes can also function as B-cell helpers [45]. They induce, indeed, a pronounced antibody response, with preferential immunoglobulin (Ig) class switch to IgG2a and IgG3 for IL-17 and to IgG1 and IgG2b for IL-21. These results establish that Th17 cells are crucial in germinal center (GC) formation. As suggested above, the proinflammatory IL-17, normally considered a T-cell-associated factor, has been also reported to be a central driver of GC-derived autoantibodies. This was demonstrated by blocking IL-17 signaling that disrupted the CD4^+^T-cell and B-cell interactions required for GC formation [46].

The evidence that IL-17-knockout (KO) mice are less prone to develop autoimmune diseases such as type 1 diabetes, collagen-induced arthritis, and experimental autoimmune encephalomyelitis [41, 47] raised the hypothesis that this cytokine, and therefore IL-17-producing cells, may also be involved in the pathogenesis of pSS. Experimental SS in the IL-17-KO mouse was only recently evaluated [48]. Despite being immunized with salivary gland peptides, these animals did not develop any histological signs of salivary gland inflammation. Intriguingly, the adoptive transfer of Th17 cells was able to induce a focal sialadenitis similar to that of wild type mice, underscoring the pathogenic role of IL-17 also in pSS. It is of note, that already in 2008, Nguyen et al. first reported that IL-17 overexpression in salivary glands by adenovirus vectors was able to trigger a SS-like condition in nonsusceptible mice [49]. In this setting, three studies described that IL-17 is consistently expressed in the periductal infiltrates of all MSGs from patients with pSS [50–52] and in two of them such expression was found to be associated with the severity of glandular inflammation [51, 52].

In addition, most of the cytokines that support the Th17 phenotype as well as other Th17-cell products, including IL-6, IL-21, IL-22, and IL-23 and their receptors, are consistently expressed in MSGs of patients with pSS [50, 52–57]. Similar to IL-17, also IL-21 expression in MSGs appears to parallel the severity of glandular inflammation [53].

The main cellular source of glandular IL-17 in pSS-MSGs appears to be constituted by CD4^+^ and, to a lesser extent, CD8^+^ T lymphocytes [58, 59]. Of interest, however, two recent studies pointed out that also CD4^−^CD8^−^ (double negative, DN) T cells [60, 61] and mast cells [55] may participate in IL-17 local balance in pSS.

DN T cells were initially identified as a source of IL-17 in SLE [62] and their presence in the inflammatory infiltrate of kidney during lupus nephritis suggested a pathogenic role of this cell subset in SLE. In pSS, DN T cells were found to be associated not only with the extent of glandular involvement, as already demonstrated for IL-17 [51, 52], but also with the presence of MSG ectopic lymphoid structures, which have been associated with more severe clinical phenotype, including B-cell lymphoma [7, 63]. This evidence appears to support the pathogenic role of IL-17 and IL-17-producing cells not only in the induction but also in the perpetuation of glandular lesions.

IL-17 and its related cytokines have also been evaluated in other biological samples from pSS patients such as saliva, tears, and serum. To date, only one study assessed IL-17 in pSS saliva reporting higher levels of this cytokine compared to non-pSS, but failing to identify any association between the concentration of this cytokine and the extent of MSG lymphocytic infiltration [64].

In addition, there is general agreement among available studies of increased IL-17 concentration in the tears of pSS patients compared to non-pSS dry eye [65–68].

As far as serum is concerned, the four studies that assessed IL-17 pointed out that only a subgroup of pSS patients display detectable levels of this cytokine [50, 51, 69, 70], but only two of these found an association between serum IL-17 and clinical/histological features of pSS [69, 70]. In particular, it was shown that disease duration was significantly longer and parotid gland swelling was less prevalent in IL-17-positive patients compared to those IL-17-negative ones. However, multivariate analysis revealed that disease duration was associated with the presence of serum IL-17 independently of concurrent parotid gland swelling [70]. Furthermore, IL-17 serum concentration was higher in patients with ectopic GC-like structures compared to those without GCs [69].

Also IL-21 has been found to be increased in pSS serum [53], while IL-6 and IL-23 have been found to be increased in pSS plasma in association with increased levels of IL-17 [52].

To shed additional light on peripheral IL-17 balance, enumeration of IL-17-producing cells in the PB has been performed in a series of studies. An overall increase of circulating CD4^+^Th17 cells [51, 59–61] and DN T cells [60, 61] has been described in pSS patients compared to controls. Intriguingly, however, in a study performed by our group, differences in the percentage of these cell subsets according to disease duration were also highlighted. It appeared, indeed, that in early pSS, with symptom duration less than 18 months, CD4^+^Th17 cells were expanded, while DN T cells were comparable to those of normal subjects. Conversely, in patients with established disease and symptom duration over 5 years, CD4^+^Th17 cell percentage was comparable to that of controls and DN T cells were expanded [61].

Taken together, these findings in pSS MSG, serum/plasma, and PB suggest a highly dynamic scenario occurring in the course of the disease with a clear pathogenic role of IL-17 in triggering and maintaining glandular inflammation and a recirculation of IL-17-producing T-cell subsets from PB to MSG and vice versa in different phases of the disease (Table 1). However, despite their clear pathogenic role, no association between IL-17/Th17 cells and severity of clinical picture or specific extraglandular clinical manifestations has been reported [71].

We believe that this last consideration does not diminish but rather reinforces the role of IL-17/Th17 system in pSS pathogenesis. In fact, this biological system is crucial in the induction as well as in the maintenance of pSS, independently of the clinical or serological features of the disease, thus representing an interesting and promising therapeutic targeting in this disease.

In conclusion, although interesting, all the aforementioned studies evaluating Treg and Th17 cells are weighted by a number of intrinsic limitations that deserve some consideration. First, pSS is a heterogeneous disease characterized by different genetic background (e.g., HLA haplotypes), clinical manifestations (glandular versus extraglandular), and serological status (anti-SSA, anti-SSB, both, or none). All the above imply different therapeutic approaches (lacrimal or salivary substitutes versus immune suppressants). Since in most studies patients are not subgrouped according to disease features, including disease duration or therapy, results may be biased. Furthermore, the majority of investigations are performed in peripheral blood rather than target organs providing a partial view on such scenario. Therefore, the balance of different cytokines and soluble mediators as well as their effects on T cells should be investigated* in vivo* rather than* in vitro*. Finally, it is still a matter of debate whether Treg cell impairment and Th17 cell predominance may be either a cause or a consequence of the ongoing inflammation.

## 4. Therapeutic Perspectives

pSS is an autoimmune disorder affecting exocrine glands and is characterized, in most cases, by a rather mild clinical picture. However, a subgroup of pSS patients experience systemic extraglandular involvement leading to a worsening of disease prognosis. Current therapeutic options for the treatment of pSS are mainly empiric, often translated by other autoimmune diseases, and recent systematic reviews highlighted the lack of evidence-based recommendations for most of the immunosuppressive drugs commonly employed in the spectrum of extraglandular involvement. In this setting, therefore, pSS may be still considered an orphan disease [72–75].

In recent years, the effects of currently available therapies on Treg and Th17 cells have been extensively investigated in autoimmune diseases, in particular RA and psoriasis [40, 76]. Unfortunately, however, very few data concerning this issue are available in pSS. This is partially explained by the fact that only a subgroup of patients display extraglandular manifestations requiring immunosuppressive therapies. Furthermore, most of the biologic therapies employed in RA are not currently used in pSS for the lack of either proven clinical efficacy (e.g., TNF blockers), clinical trials (e.g., tocilizumab), or, although effective, specific licensing (e.g., abatacept).

It is of note, however, that corticosteroids (CS) may affect the IL-17 axis during pSS. Although a case report described infiltrate reduction in one patient with pSS receiving CS at high doses, no further evidence supports the efficacy of CS in reducing glandular inflammation to date [77, 78]. Subsequently, it has been demonstrated that the subset of IL-17-producing DN T lymphocytes, isolated from pSS patients, is resistant to CS* in vitro* [60]. Conversely, DN T cells from normal controls promptly respond to CS* in vitro* by reducing IL-17 production. These observations give rise to an intriguing line of investigation in the clinical scenario of CS-resistance of autoimmune/inflammatory diseases.

As far as Treg cell selective therapeutic targeting is concerned, approaches promoting the* in vivo* expansion of Tregs or injection of* in vitro* expanded autologous/heterologous Tregs are under intense investigation in different acute and chronic diseases to date with promising results. In particular, data from murine studies suggest that the transfer of autologous Treg cells is able to prevent the development of collagen-induced arthritis and colitis, while* ex vivo* expanded CD4^+^CD25^+^ Treg cells can attenuate the development of experimental autoimmune encephalomyelitis and ameliorate type I diabetes [35]. In humans, Treg cells transfer is able to prolong immune tolerance following hematopoietic stem cell transplantation and to prevent graft-versus-host disease [79]. Treg cell transfer is safe in patients with severe Crohn's disease [80] and an open-label phase I trial to investigate safety and efficacy of intravenous infusion of* ex vivo* selected and expanded autologous polyclonal Treg cells in patients with type 1 diabetes is currently ongoing (trial NCT01210664). These encouraging results allow speculating that reprogramming Treg cells or infusing* in vitro* expanded autologous/heterologous Treg cells may affect the natural history of autoimmune diseases [81]. However, two main concerns limit the possible employment of Treg cell therapy. First, taken the aforementioned debate about the specificity of CD25 as Treg cell marker, additional molecules should be identified to isolate and expand the most suitable Treg cell subset. In this setting, since under some conditions, the regulatory activity of CD4^+^CD25^high^GITR^+^ cells is superior to that of CD4^+^CD25^high^GITR^−^ cells, GITR may be a good candidate [35].

Second, stability and fate of transferred Treg cells* in vivo* are not predictable and, according to recent findings, they may be converted into effector cells, namely, Th17 cells, by proinflammatory cytokines [16, 82].

On the other hand, the growing amount of data supporting the pathogenic role of IL-17 axis in the pathogenesis of pSS provided the rational for a number of clinical trials that are currently ongoing in pSS.

Two monoclonal antibodies against IL-17 (the fully human IgG1k secukinumab and the humanized IgG4 ixekizumab) are being investigated in inflammatory arthritides [83] and a clinical trial evaluating secukinumab in dry eye patients is currently ongoing (trial NCT01250171).

Concerning the molecules that are involved in the balance between Treg and Th17 cell commitment, the humanized anti-IL-6 antibody tocilizumab is employed in clinical practice for the treatment of RA and a phase II trial to assess its efficacy in pSS is currently ongoing (trial NCT01782235). Since IL-6 presence or absence in the local microenvironment drives the polarization of a naïve T cell to a Th17 or a Treg phenotype, respectively, it would be of great interest to ascertain the effect of IL-6 blockade in such a scenario.

Compounds targeting IL-23 and IL-21 have been or are under investigation in plaque psoriasis and chronic inflammatory arthritides, but not yet in pSS [84, 85].

Another interesting aspect is the possible effect on IL-17 axis exerted by non-T-cell selective compounds. In fact, previous studies that investigated the effects of anti-CD20 antibody rituximab on T lymphocytes in RA revealed that this compound is able to deplete CD4^+^Th17 cells in PB and synovium as they coexpress CD20 [86–88]. Taken that rituximab is currently the most employed biologic agent in pSS [89], if these observations will be confirmed in the PB and MSG of pSS patients, an additional rational for therapeutic application of rituximab in pSS may be defined.

Finally, another intriguing approach is represented by the interference with Th17 commitment via the modulation of ROR*γ*t activity in the thymus. A study that employed a synthetic ligand binding to this transcription factor in a mouse model of multiple sclerosis reported impressive clinical efficacy [90].

These data point out the need of randomized clinical trials to investigate the safety and efficacy of these drugs in pSS in order to provide solid scientific evidence. Taken together, the difficulty to build therapeutic recommendations in pSS may be related to the heterogeneity of clinical picture, the frequent failure of first line treatments, the lack of scientific evidence for drugs licensed for other diseases, and, finally, the lack of innovative therapeutic compounds.

## 5. Conclusion

pSS encompasses several subsets of patients with different genetic background, pathophysiological pathways, demographic features, and different response to proposed therapies. Despite the acknowledged role of T-cell subsets in pSS, mechanisms leading to their abnormal activation and their contribution to pSS pathogenesis are not fully elucidated. In particular, only few studies evaluated the role of Treg cells in pSS, and conclusive data are still lacking. Similarly, although conventional CD4^+^Th17 and IL-17-producing DN T cells are under active investigation, their origin, fate, and function are still a matter of debate. Currently available data suggest a pivotal role of IL-17 axis and IL-17-producing cells in every step of pSS pathogenesis, from the induction of autoimmune epithelitis to the maintenance and perpetuation of inflammation and ectopic GC formation. In this context, IL-17-producing T cells seem to be a link between T- and B-cell compartments. In this setting, a therapeutic approach targeting IL-17 axis may represent an intriguing issue worth to be investigated in pSS. In the era of biologic agents, randomized double-blind controlled trials represent the most powerful tool to obtain comparable results and provide the rationale to build solid therapeutic recommendations also in pSS.

## Figures and Tables

**Table 1:** Indicators of Treg cell and IL-17/IL-17-producing T cell involvement in primary Sjögren's syndrome.

Source	Observation	References
Salivary glands	FoxP3 expression is associated with the severity of glandular inflammation Inverse relationship between glandular and circulating FoxP3+ cells Lower FoxP3+ cells correlate with adverse predictors for lymphoma development CD25low/-GITR+ T cells are present in mononuclear cell infiltrate High expression of IL-17 and IL-17R High expression of IL-6, IL-21, IL-22, and IL-23 CD4+, CD8+, mast cells, and DN T cells produce IL-17 IL-17 is associated with the severity of glandular damage DN T cells are associated with GCs	[21, 23] [21] [21] [26] [50–52] [50, 52–57] [55, 60, 61] [51, 52] [61]

Saliva/tears	Increased levels of IL-17 in saliva Increased levels of IL-17 in tears No association between salivary IL-17 and glandular damage	[64] [65–67] [64]

Serum	Increased levels of IL-17 Increased levels of IL-6, IL-21, and IL-23 IL-17 prevalence is dependent on disease duration Serum IL-17 is higher in patients with MSG-GCs	[50, 51, 69, 70] [52, 53] [70] [69]

Peripheral blood	Altered Treg cell percentage (increased, decreased, or comparable percentages with respect to HD) Inverse correlation between the percentage of Treg cells and CRP, ESR, RF, and IgG No differences in Treg cell percentage according to ESSDAI and SSDAI Expansion of functionally suppressive CD25low/-GITR+T cells Suppressive CD25low/-GITR+T cell percentage are expanded in inactive pSS Increase of circulating CD4+Th17 cells Increase of circulating IL-17+DN T cells	[17–26] [19] [25, 26] [26] [26] [26] [51, 59–61] [60, 61]

Intrinsic cell abnormalities	The Vbeta repertoire of pSS Treg cells is polyclonal and not significantly restricted as compared with that in controls IL-17-producing DN T cells are totally insensitive to dexamethasone *in vitro* IL-17A gene displays an association with GC status	[17] [60] [91]

Treg = T regulatory cells; IL = interleukin; Th17 = IL-17-producing T helper cells; FoxP3 = Forkhead box protein P3; GITR = glucocorticoid-induced tumor necrosis factor receptor related protein; DN = IL-17-producing double negative T cells; GC = germinal center; CRP = C reactive protein; ESR = erythrocyte sedimentation rate; RF = rheumatoid factor; IgG = immunoglobulin G; ESSDAI = EULAR Sjögren's syndrome disease activity index; SSDAI = Sjögren's syndrome disease activity index; and HD = healthy donors.
